# DA-9805, a Herbal Mixture, Restores Motor Manifestations in 6-Hydroxydopamine-induced Parkinson’s Disease Mouse Model by Regulating Striatal Dopamine and Acetylcholine Levels

**DOI:** 10.3389/fphar.2022.903664

**Published:** 2022-06-15

**Authors:** Eugene Huh, Youngji Kwon, Jin Gyu Choi, Myung Gyu Lim, Jin Seok Jeong, Ah Yeon Park, Jeong Hee Kim, Youngmi Kim Pak, Seon-Pyo Hong, Myung Sook Oh

**Affiliations:** ^1^ Department of Oriental Pharmaceutical Science, College of Pharmacy, Kyung Hee University, Seoul, South Korea; ^2^ Department of Life and Nanopharmaceutical Sciences, Graduate School, Kyung Hee University, Seoul, South Korea; ^3^ Department of Oriental Pharmaceutical Sciences, Graduate School, Kyung Hee University, Seoul, South Korea; ^4^ R&D Center of Dong-A ST, Yong-in, South Korea; ^5^ Medical Research Center for Bioreaction to Reactive Oxygen Species and Biomedical Science Institute, Department of Physiology, College of Medicine, Kyung Hee University, Seoul, South Korea; ^6^ Department of Biochemical and Pharmaceutical Sciences, Graduate School, Kyung Hee University, Seoul, South Korea

**Keywords:** DA-9805, Parkinson’s disease, striatal neurotransmission, dopamine, acetylcholine, homeostasis

## Abstract

Loss of dopamine (DA) is one of the primary features of Parkinson’s disease (PD); however, imbalances of non-dopaminergic neurotransmitters significantly contribute to the disabilities noted in advanced PD patients. DA-9805 is the ethanolic extraction of the root bark of *Paeonia* × *suffruticosa* Andrews (*Paeoniaceae*), the root of *Angelica dahurica* (Hoffm.) Benth. and Hook.f. ex Franch. and Sav. (*Apiaceae*) and the root of *Bupleurum falcatum* L. (*Apiaceae*), which have been widely utilized as an enhancer of motor function in East Asia. This study aimed to investigate whether DA-9805 modified motor dysfunctions and imbalances associated with DA and other neurotransmitters in a 6-hydroxydopamine-induced PD mouse. We confirmed the expressions of proteins related with neurotransmissions in the striatum. In addition, we measured the striatal neurotransmitters using HPLC and analyzed their correlation. DA-9805 significantly improved motor impairments and restored the altered levels of neurotransmitters in the striatum. Moreover, DA-9805 improved the altered expressions of tyrosine hydroxylase (TH), DA transporter, and choline acetyltransferase (ChAT) in the ipsilateral part of mouse striatum or SNpc, which implies the neuroprotection. We also found that the level of striatal acetylcholine (Ach) has the moderate negative correlation with motor functions and TH expression in the SNpc. This study indicates that DA-9805 restores motor dysfunctions by normalizing the increased levels of striatal Ach via modulating DA transmission and ChAT expressions as well as its neuroprotective effects.

## 1 Introduction

Parkinson’s disease (PD) is a progressive neurodegenerative disease that is pathologically characterized by the death of dopaminergic neurons projecting from the substantia nigra pars compacta (SNpc) to the striatum (ST) (Alexander, 2004). Loss of dopamine (DA) in the ST that exceeds 60–80% has been associated with the motor symptoms of PD, which include bradykinesia, resting tremor, rigidity and instability of posture (Dauer and Przedborski, 2003). Despite the fact that the etiology of neuronal loss in PD requires further elucidation, it has been reported in the pathophysiology of PD as part of the alteration in neurotransmitters, excitotoxicity, oxidative stress, mitochondrial dysfunction and neuroinflammation (Sun et al., 2012; Jamwal and Kumar, 2019).

ST is the largest structure of the basal ganglia (BG), and has an important function of controlling the voluntary movements. Studies have reported the presence of projection neurons (medium spiny neurons and interneurons) that contain several neurotransmitters including DA, glutamate (Glu), γ-aminobutyric acid (GABA), and acetylcholine (Ach) in ST (Jamwal and Kumar, 2019). DA deficiency in the ST has been associated with the disruption of other neurotransmitters, which has subsequently been attributed to the occurrence of neurological disorders including PD (Sun et al., 2012; Ztaou et al., 2016; Jamwal and Kumar, 2019).

Neurotransmitters that are an essential part of the BG circuit are activated by Glu, and depressed by GABA; furthermore, the activation of these two systems is relevant in the pathophysiological conditions of PD (Luscher et al., 2011). Glutamatergic projections subsequently become overactive and increase striatal Glu with the loss of DA in the nigrostriatal pathway (Blandini et al., 1996; Eo et al., 2019). However, GABA deficiency may manifest in both experimental PD animal models and bradykinesia of PD patients (Jamwal and Kumar, 2019). Striatal Ach release would lead to movement control by inversely interacting with DA. Additionally, increase in Ach levels has been observed in PD patients and the associated symptoms have been relieved by the prescription of anticholinergic drugs (Bohnen and Albin, 2011). Therefore, it may be potentially used to develop PD therapeutic modalities to regulate the release of DA and other neurotransmitters.

DA-9805 is a mixture of 90% ethanol extracts of Moutan Radicis Cortex [MRC; the root bark of *Paeonia* × *suffruticosa* Andrews (*Paeoniaceae*)], *Angelica dahuricae* Radix [ADR; the dried root of *Angelica dahurica* (Hoffm.) Benth. and Hook.f. ex Franch. and Sav. (*Apiaceae*)], and Bupleuri Radix [BR; the root of *Bupleurum falcatum* L. (*Apiaceae*)]. These herbal medicines are the representative traditional medicines which have been widely utilized as a enhancer of motor function by smoothing muscle and stimulating autonomic nervous systems in East Asia (Lee et al., 2009; Küpeli Akkol et al., 2021). In an aspect of pharmacological researches, MRC is considered to include bioactive compounds such as peaonol, paeoniflorin, oxyparoniflorin, garlic acid, and peaoniflorigenone (Kwon et al., 2017). Studies have reported that MRC attenuated 1-methyl-4-phenyl-1,2,3,6-tetrahydropyridine (MPTP)-induced striatal DA loss (Kim et al., 2014). ADR contains major bioactive compounds such as imperatorin, isoimperatorin, oxypeucedanin, phellopterin, and byakangelicol (Kwon et al., 2017). ADR inhibited GABA transaminase activities (Choi et al., 2005; Cao et al., 2017). Imperatorin has reportedly increased serotonin concentration in brain (Cao et al., 2017). BR primarily includes bioactive compounds such as saikosaponins (Eo et al., 2019) Maccinoi et al. reported that oral administration of saikosaponin A reduced addiction–like behaviors *via* a GABAB receptor–mediated mechanism Maccioni et al. (2016).

The neuroprotective effects of DA-9805 against neurotoxins MPTP or 6-hydroxydoapmine (6-OHDA) has previously been demonstrated in experimental PD models (Jeong et al., 2018; Eo et al., 2019). However, there is a need to elucidate the therapeutic effects of administering DA-9805 post-treatment for PD symptoms, by regulating the levels of striatal neurotransmitters in 6-OHDA induced PD mice model. Here, we assessed performance in behavioral tests after inducing 6-OHDA toxicity, followed by DA-9805 administration after a period of 1 week. Additionally, we used a combination of high-performance liquid chromatography and integrated pulsed amperometric detection (HPLC-IPAD) analysis to explore the involvement of DA-9805 in the alterations associated with striatal neurotransmitters. We subsequently analyzed the correlation between the striatal neurotransmitters and motor symptoms to identify the specific striatal neurotransmitters that regulate DA-9805–associated actions. Furthermore, we observed the histological changes in regulatory factors that result in the contents of neurotransmitters.

## 2 Materials and Methods

### 2.1 Materials

6-OHDA, ascorbic acid, 3,4-Dihydroxy-L-phenylalanine (L-DOPA), benserazide, paraformaldehyde (PFA), hydrogen peroxide, 3,3-diaminobenzidine (DAB), sucrose, tribromoethanol, bovine serum albumin (BSA), tribromoethanol, phosphate buffered saline (PBS), and standard compound for HPLC were purchased from Sigma Aldrich (St Louis, MO, United States). Lanatoside was purchased from ChromaDex (Irvine, CA, United States) and was used for internal standard (I.S.). HPLC-grade trifluoroacetic acid (TFA) was purchased from DAEJUNG (Gyeonggi-do, South Korea). 50% sodium hydroxide (NaOH) were purchased from Fisher Scientific (Fairlawn, NJ, United States). Rabbit anti-tyrosine hydroxylase (TH; cat.no. AB152), rat anti-dopamine transporter antibody (DAT; cat.no. MAB369), anti-choline acetyltransferase (ChAT; cat.no. AB144P) were purchased from Merck Millipore (Burlington, MA, United States). Biotinylated goat anti-rabbit antibody, avidin–biotin complex (ABC), normal goat serum, streptavidin-Alexa 594 and chicken anti-goat Alexa 488 were purchased from Vector Labs (Burlingame, CA, United States). Tween 20, ammonium persulfate, acrylamide, enzyme-linked chemiluminescence reagent, and skimmed milk were purchased from Bio-Rad Labs (Hercules, CA, United States). HRP-conjugated anti-β-actin antibody (cat.no. SC-47778HRP) was purchased from Santa Cruz Biotechnology (Dallas, TX, United States). Radio-immunoprecipitation assay (RIPA) buffer and protease/phosphatase inhibitor cocktail were purchased from Thermo Fisher Scientific (Waltham, MA, United States).

### 2.2 Preparation of DA-9805

A standardized DA-9805 (batch number: MB1603) was provided from Dong-A ST (Yong-in, South Korea). Condition for extraction and quality control of DA-9805 were described as reported methods previously (Jeong et al., 2018). Briefly, same amounts of MRC, ADR, and BR were extracted at room temperature (20–25°C) for 24 h with 90% ethanol, evaporated and subsequently maintained at 4°C until use. The reproducibility of DA-9805 was confirmed by ultra-HPLC fingerprinting validation analysis as reference.

### 2.3 Animals

ICR mice (male, 6-week-old, 27–30 g) were purchased from Daehan Biolink Co., Ltd. (Eumseong, South Korea). Mice were accommodated at a maintained condition (temperature: 23 ± 1°C, humidity: 60 ± 10% a 12 h light/dark cycle, and water and food ad libitum). All of the experiments were performed in accordance with the National Institute of Health Guide for the Care and Use of Laboratory Animals (NIH Publications No. 80-23) revised 1996 and with protocols from the Institutional Animal Care and Use Committee of Kyung Hee University [approved number: KHUASP (SE)-18-114].

### 2.4 Surgical Procedure for Injection of 6-OHDA

Intrastriatal injection of 6-OHDA was performed for each mouse based on the methods described in a previous study (Huh et al., 2018). Essentially, each mouse was anesthetized with tribromoethanol followed by a unilateral injection of 2 μl vehicle (saline with 0.1% ascorbic acid for sham-operated mice) or 16 μg of 6-OHDA in 2 μl vehicle into the target region (coordinates with respect to bregma in mm: AP 0.5, ML 2.0, DV−3.0), according to the stereotaxic atlas of mouse brain (Franklin, 2013) on a stereotaxic apparatus (myNeuroLab, St. Louis, MO, United States). Vehicle and 6-OHDA were injected by a microinjection pump at an injection rate of 0.5 μl per minute, and the cannula was left in place for 4 min after the end of injection.

### 2.5 Experimental Design

#### 2.5.1 Experiment 1

36 mice were used in the 1st experiment. It was aimed to determine the dose-dependency of effects of DA-9805 on motor manifestations in 6-OHDA-induced PD mice and utilized for histological analysis. Mice were randomly divided into 6 groups as follows; 1) Sham group (Vehicle-lesioned plus vehicle-treated group, *n* = 6), 2) 6-OHDA group (6-OHDA-lesioned plus vehicle-treated group, *n* = 6), 3) L-DOPA (6-OHDA-lesioned plus 50 mg/kg of L-DOPA with 12.5 mg/kg benserazide-treated group, *n* = 6), 4) DA-9805 1 (6-OHDA-lesioned plus 1 mg/kg of DA-9805-treated group, *n* = 6), 5) DA-9805 3 (6-OHDA-lesioned plus 3 mg/kg of DA-9805-treated group, *n* = 6) and 6) DA-9805 10 (6-OHDA-lesioned plus 10 mg/kg of DA-9805-treated group, *n* = 6).

#### 2.5.2 Experiment 2

32 mice were used in the 2nd experiment. It was for the additional biochemistry analysis of neurotransmissions. Mice were randomly divided as follows; 1) Sham group (Vehicle-lesioned plus vehicle-treated group, *n* = 8), 2) 6-OHDA group (6-OHDA-lesioned plus vehicle-treated group, *n* = 12), 3) DA-9805 10 (6-OHDA-lesioned plus 10 mg/kg of DA-9805-treated group, *n* = 12).

#### 2.5.3 Drug Preparation and Treatment

DA-9805 or L-DOPA were dissolved in the normal saline as a vehicle. L-DOPA was treated with benserazide simultaneously as a suspension (Dauer and Przedborski, 2003; Alexander, 2004). In the all experiments, drugs or vehicle were administered in each mouse using gavage for 10 days from the 7th day after surgery. Drugs and vehicle administrations were conducted once per day. During the period of behavior tests, drugs and vehicle were administered 2 h before the assessments (Sun et al., 2012; Jamwal and Kumar, 2019).

### 2.6 Assessment of Motor Functions

#### 2.6.1 Pole Test

We performed the pole test at the 15th day of post-surgery. The mice were positioned with their head facing upward on top of the pole (diameter 8 mm, height 55 cm, with a rough surface). The time required to turn down (T-turn) and to land down (T-LA) was recorded.

#### 2.6.2 Rotarod Test

The rotarod test was carried out at the 16th day of post-surgery. The rotarod unit included a rotating spindle (7.3 cm diameter) and five individual compartments to simultaneously examine 5 mice. Day before the examination, the 15th day of 6-OHDA injection, mice in all groups were trained on the rotarod apparatus for 180 s for three times. Rotarod test was performed at a constant speed that varied between 4–6 rpm. Training sessions were performed by putting the mice on top of the rod every time they fell into the ground. Resting time between trials was at least 30 min. During the test session, we conducted it same as training trials, whereas rotating speed was 6–8 rpm. The duration for which each mouse remained on the rotating rod until the first fall (latency time) and number of frequent falls from the rod for 180 s were recorded.

#### 2.6.3 Apomorphine-Induced Rotation Test

Apomorphine-induced rotation test was accomplished at the 16th day after 6-OHDA lesion. Mice were placed in a hemispheric rotational bowl (40 diameter) and were habituated to adjust environment for 5 min before apomorphine administration (4 mg/kg, s.c.). Full 360° turns in the direction opposite to the lesion (contralateral rotation) were counted for 25 min.

### 2.7 Tissue Preparation

Seventeen days after the surgery, each mouse was sacrificed as follows; for immunohistochemistry or immunofluorescence analysis (Experiment 1), mice were anesthetized and perfused transcardially with 0.05 M phosphate buffer saline and subsequently fixed with pre-chilled 4% paraformaldehyde in 0.1 M phosphate buffer. Then whole brain tissues were post-fixed with 4% paraformaldehyde for overnight at 4°C. For western blot and HPLC analysis (Experiment 2), mice were sacrificed by cervical dislocation. ST and SN regions from brains were dissected in accordance to the mouse atlas (Franklin, 2013).

### 2.8 Immunohistochemistry

We used a freezing microtome to acquire serial 30 μm-thick coronal sections (Leica Instruments GmbH, Nussloch, Germany) and then stored them in a cryoprotectant (25% ethylene glycol, 25% glycerol, and 0.05 M phosphate buffer) at 4°C until use. The floating brain sections including ST region (AP 0–0.5 mm from bregma) were incubated overnight with a rabbit anti-TH (1:1000), a rat anti-DAT antibody or a goat anti-ChAT antibody (1:500), after reacted with 1% hydrogen peroxide. The sections including SNpc regions (AP −3.3 to −3.7 mm from bregma) were incubated overnight with a rabbit anti-TH (1:1000) antibody, as a same protocol for ST. They were subsequently incubated with a biotinylated anti-rabbit IgG (1:200), anti-rat IgG or anti-goat IgG followed by being incubated in an ABC solution. DAB was used to develop the color of every section and the images were photographed using an optical light microscope (Olympus Microscope System BX51; Olympus, Tokyo, Japan). The ChAT-immunopositive neurons in the dorsal ST and the TH-immunoreactive neuronal cells in the SNpc at a ×100 magnification; furthermore, the optical density of TH-positive or DAT-positive fibers was measured in the dorsal ST at a ×40 magnification using ImageJ software (National Institutes of Health, Bethesda, MD, United States). ChAT and TH- positive neuronal cell counts were done by stereological analyses. The optical density and cell counts were conducted by an experimenter who was blinded to the treatment condition, and the outcome for each animal was a mean of its results from the three sections.

### 2.9 Immunofluorescence

Immunofluorescence was performed by rinsing the brain tissues (AP 0–0.5) in PBS, followed by being incubated overnight with a rabbit anti-TH (1:1000) and a goat anti-ChAT antibody (1:500 dilutions). They were subsequently incubated with a Alexa FluorTM 488 chicken anti-goat IgG antibody (1:500 dilutions) and followed by the incubation with a DyLightTM 594 goat anti-rabbit IgG antibody (1:500 dilutions). The representative images were visualized using confocal microscopy at a ×40 and ×100 magnification (K1-Fluo, Nanoscope Systems, Daejeon, Korea).

### 2.10 Western Blot Analysis

Tissues were lysed with a RIPA buffer containing protease/phosphatase inhibitors for whole protein analysis. The lysates were then conducted as described in the previous experiment (Park et al., 2013). We quantified the protein concentrations using Bradford’s assay. Each sample was subsequently separated on 10% SDS gels, transferred to a membrane, and blocked with 5% skim milk in TBST. Membranes were incubated with rabbit anti-TH and HRP-anti-β-actin (1:2000 dilutions) antibody for overnight at 4°C and then incubated with HRP-conjugated rabbit IgG for 1 h. Immunoreactive bands were developed using an enzyme-linked chemiluminescence detection kit and visualized using the ChemiDocTM XRS + system (Bio-rad, Hercules, CA, United States).

### 2.11 Measurement of Neurotransmitter Levels by HPLC

#### 2.11.1 Sample Preparation

Contents of the neurotransmitters were measured by preparing the samples of the 6-OHDA-lesioned regions of the ST. ST was homogenized in 0.2 M HClO4 and then were centrifuged for 10 min at 4°C with 12,000 g. The supernatant was filtered through a 0.20 μm membrane filter, and transferred to the HPLC machine. The number of biogenic amines and their metabolites were expressed as “ng/mg” of the total protein. The Bradford’s protein assay was used to quantify the amount of proteins.

#### 2.11.2 Neurotransmitters

HPLC equipment, which included the 3201 Dual Pump and Nanospace SI-2/3001 pump, was purchased from Shiseido Co. (Tokyo, Japan). The amount of DA in the mouse ST (N = 4 per group) was measured by a method described in a previous study (Oh et al., 2018).

For other neurotransmitters (N = 4 for the Sham group and N = 8 per 6-OHDA and DA-9805 10 group), the PAD from the ICS-3000 series Dionex (Sunnyvale, CA, United States) was equipped with an Au-Flow cell containing a gold working electrode, stainless steel auxiliary electrode, and solvent-compatible cell containing an Ag/AgCl reference electrode. The following six-potential waveform was used: E1 = −0.20 V (0.00–0.04 s); E2 = 0.00 V (0.05–0.21 s); E3 = 0.22 V (0.22–0.46 s); E4 = 0.00 V (0.47–0.56 s); E5 = −2.00 V (0.57–0.58 s); and E6 = 0.60 V (0.59 s).

A PC HILIC column (250 × 4.6 mm I.D; 5 µm Shiseido Co, Tokyo, Japan) was used to perform the chromatographic separation using an isocratic elution program. The mobile phase included 0.1% trifluoroacetic acid, and the flow rate was set to 1 ml/min, while the column temperature conditions were maintained at room temperature. A post-column delivery system composed of 200 mM NaOH with a flow-rate of 0.8 ml/min was connected to the flow line between the column and IPAD system. A new batch of the mobile phase was made every day, and was sonicated for 30 min before use. The injection volume was 10 µl. Standard solutions were prepared using the stock solutions of each standard (ACh, Glu, and GABA). The internal standards were prepared by dissolving 1 mg of each standard in 1 ml of 20% (v/v) acetonitrile/water to achieve a final concentration of 1000 μg/ml. Information of standard was described in the Supplementary Data S1, S2. These stock solutions were stored at –4°C before analysis. The calibration curves were created for each component. The regression equation used was y = ax + b, where x and y are sample concentration and the ratios of the peak areas (components/I.S.), respectively (Supplementary Data S1, S2).

### 2.12 Statistical Analysis

Pearson’s correlation between behavior test result and levels of neurotransmitters was calculated by IBM SPSS Statistics version 13.0. Correlation analysis was performed with the mice of all groups. The rest of results through experiments were run on Prism 8.0.1 (GraphPad Software Inc., La Jolla, CA, United States). Data were analyzed by one-way analysis of variance (ANOVA) followed by Bonferroni’s multiple comparison test or Kruskal-Wallis test followed by Dunn’s multiple comparisons test in case of non-normally distributed data. All data were presented as the mean ± standard error of the mean (S.E.M.). Values were considered to be statistically significant at *p* < 0.05.

## 3 Results

### 3.1 Post-Treatment With DA-9805 Restores Motor Dysfunctions in 6-OHDA-lesioned Mice

**FIGURE 1:**
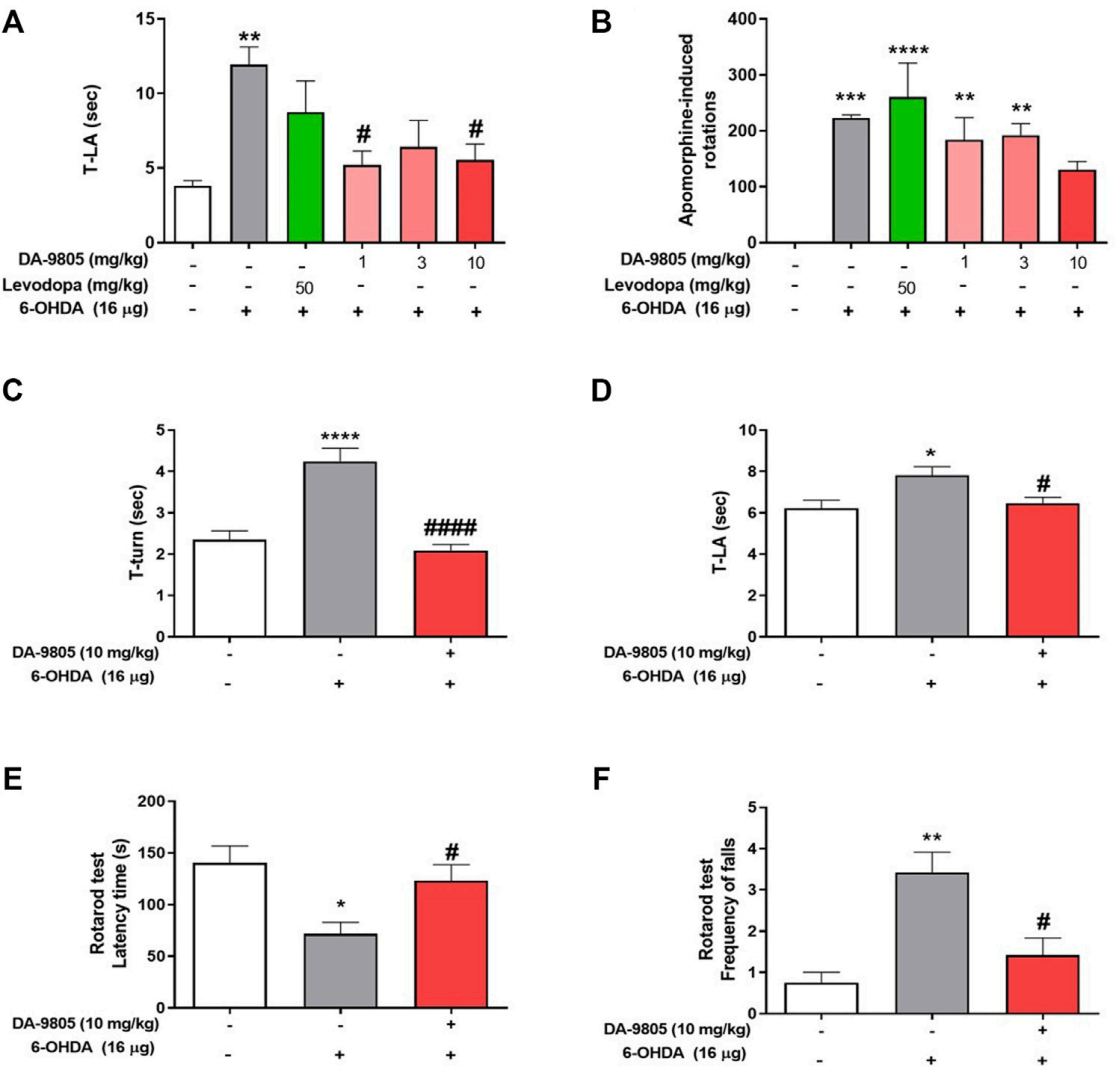
Optimal condition of DA-9805 for alleviating motor in 6-hydroxydopamine (6-OHDA)-lesioned mice. The time to arrive to the ground (T-LA) were evaluated in pole test **(A)** and net rotations induced by apomorphine for 25 min **(B)**. The time to turn (T-turn) **(C)** and T-LA **(D)** were evaluated in pole test and latency time and the frequency of falls from the rod during 180s were estimated in rotarod test **(E,F)**. Value are given as the mean ± S.E.M **(A–D)** Data were analyzed by One-way ANOVA followed by post hoc Bonferroni’s multiple comparisons test. **(E,F)** Data were analyzed by Kruskal-Wallis test followed by post hoc Dunn’s multiple comparisons test with non-normal distribution. *p < 0.05, **p < 0.01, ***p < 0.001 and ****p < 0.0001; compared to the sham group. ^#^ *p* < 0.05 and ^####^ *p* < 0.0001; compared to the 6-OHDA group. N = 6 per group.

Considering the previous reports which showed the effects of DA-9805 on motor dysfunctions and neuroprotection on PD mouse model (Eo et al., 2019), we post-treated and evaluated it’s the effect of DA-9805 on 6-OHDA-induced motor dysfunctions. To determine anti-parkinsonian effects of DA-9805 after surgery, we assessed the pole test and apomorphine-induced rotation test at 1, 3 and 10 mg/kg of DA-9805. Time to land from the pole of 6-OHDA-lesioned mice were significantly increased compared to that of the sham-lesioned mice (11.94 ± 1.19 s and 3.80 ± 0.35 s, respectively); however, mice treated with DA-9805 at 1 and 10 mg/kg (5.21 ± 0.93 s and 5.54 ± 1.07 s) significantly shorten the delayed time to descend to the ground, which were more effective than mice treated with L-DOPA (8.75 ± 2.10 s) (Figure 1A). In addition, in apomorphine-induced rotation test, DA-9805 treatment with 10 mg/kg (130.5 ± 14.51) showed a tendency to reduce turns induced by apomorphine in 6-OHDA-lesioned mice (223.5 ± 4.88, *p*-value = 0.2010), whereas, L-DOPA did not (260.33 ± 61.17) (Figure 1B). Since the 10 mg/kg of DA-9805 showed the highest improvement of motor coordinates, we determined “10 mg/kg of DA-9805” as an optimal concentration for further investigation in the current study.

Before to investigate the effects of DA-9805 on the levels of striatal neurotransmitters in 6-OHDA-lesioned mice, we confirmed its effects on motor dysfunction in the same condition. Pole test results revealed that the 6-OHDA group required a relatively greater amount of time to turn on the pole (T-turn, 4.24 ± 0.31 s) and to descend to the ground (T-LA, 7.81 ± 0.41 s) than the sham group (2.35 ± 0.21 and 6.22 ± 0.39 s, respectively). However, we noted that the DA-9805 group demonstrated significantly decreased amount of time (T-turn, 2.09 ± 0.14 and T-LA, 6.47 ± 0.28 s) (Figures 1C,D). In the rotarod test, 6-OHDA group had a relatively shorter latency time on the rod (71.79 ± 11.11 s) and an increase in the number of frequent falls (3.42 ± 0.49) from the rod than the sham group (140.38 ± 16.29 s and 0.75 ± 0.25 respectively). However, the DA-9805 group significantly improved 6-OHDA-induced motor deficits (123.25 ± 15.26 s and 1.41 ± 0.41, respectively) (Figure 1E,F).

### 3.2 DA-9805 Modifies the Alteration in the Striatal Neurotransmitter Levels Induced by 6-OHDA

**FIGURE 2:**
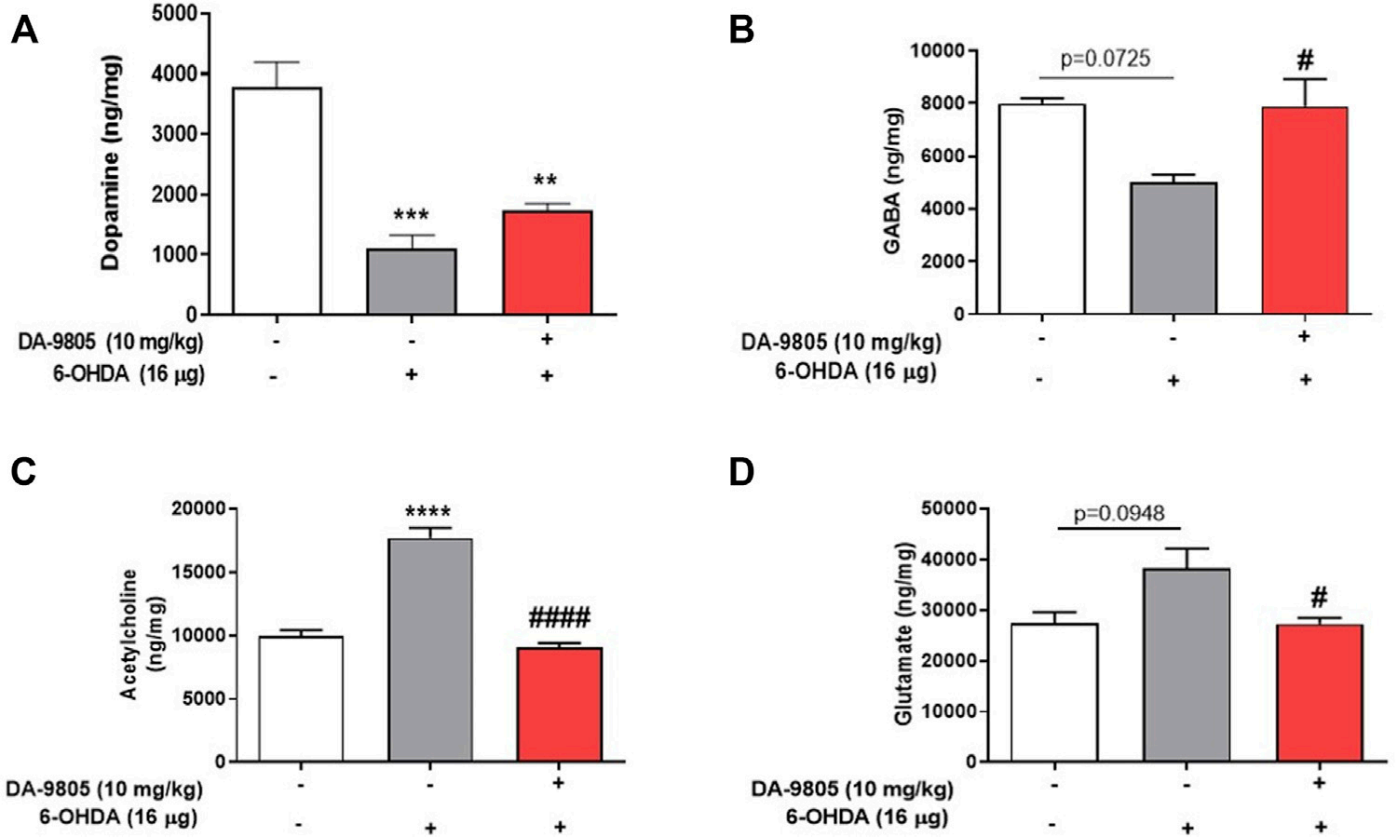
Effect of DA-9805 on levels of striatal neurotransmitters altered by 6-hydroxydopamine (6-OHDA) in mice. Striatal levels of dopamine (DA) **(A)**, gamma aminobutyric acid (GABA) **(B)**, glutamate (Glu) **(C)** and acetylcholine (Ach) **(D)**. Values are given as the mean ± S.E.M. **(A–D)** Data were analyzed by One-way ANOVA followed by post hoc Bonferroni’s multiple comparisons test. **p < 0.01, ***p < 0.001 and ****p < 0.0001; compared to the sham group. ^#^ *p* < 0.05 and ^####^ *p* < 0.0001 vs. 6-OHDA group. N = 8 in Sham group and N = 12 in 6-OHDA and DA-9805 groups.

Effects of DA-9805 on 6-OHDA-induced changes in striatal neurotransmission were measured by examining the levels of neurotransmitters in the ST. The 6-OHDA group demonstrated significantly lower levels of striatal DA (1103.72 ± 220.40 ng/mg) and GABA (5020.00 ± 282.80 ng/mg) than the sham group (DA, 3784.9 ± 414.2; GABA, 7992.00 ± 200.40 ng/mg) by 29.16 % and 62.80%, respectively. While there was no significant change in the contents of DA in the ST by DA-9805 (1741.00 ± 112.90 ng/mg, Figure 2A), DA-9805 administration altered the striatal levels of GABA (7885.27 ± 1039.00 ng/mg) compared to the those of 6-OHDA group by 157.09% (Figure 2B).

Furthermore, the striatal Ach (17733.06 ± 778.70 ng/mg) and Glu (38300.00 ± 3881.00) levels in the 6-OHDA group were higher than those of the sham group (Ach, 9998.44 ± 427.80; Glu, 27343.00 ± 2311.00 ng/mg) by 177.32 % and 140.07%, respectively; however, DA-9805 administration was associated with a significant reduction in the striatal Ach and Glu (Ach, 9053.62 ± 354.00; Glu, 27282.17 ± 1231.00 ng/mg) levels (Figures 2C,D) by 51.05 % and 71.23%, respectively, which increased by 6-OHDA.

### 3.3 DA-9805 Modulates Altered Expressions of Tyrosine Hydroxylase, Dopamine Transporter and Choline Acetyltransferase in Striatum

**FIGURE 3:**
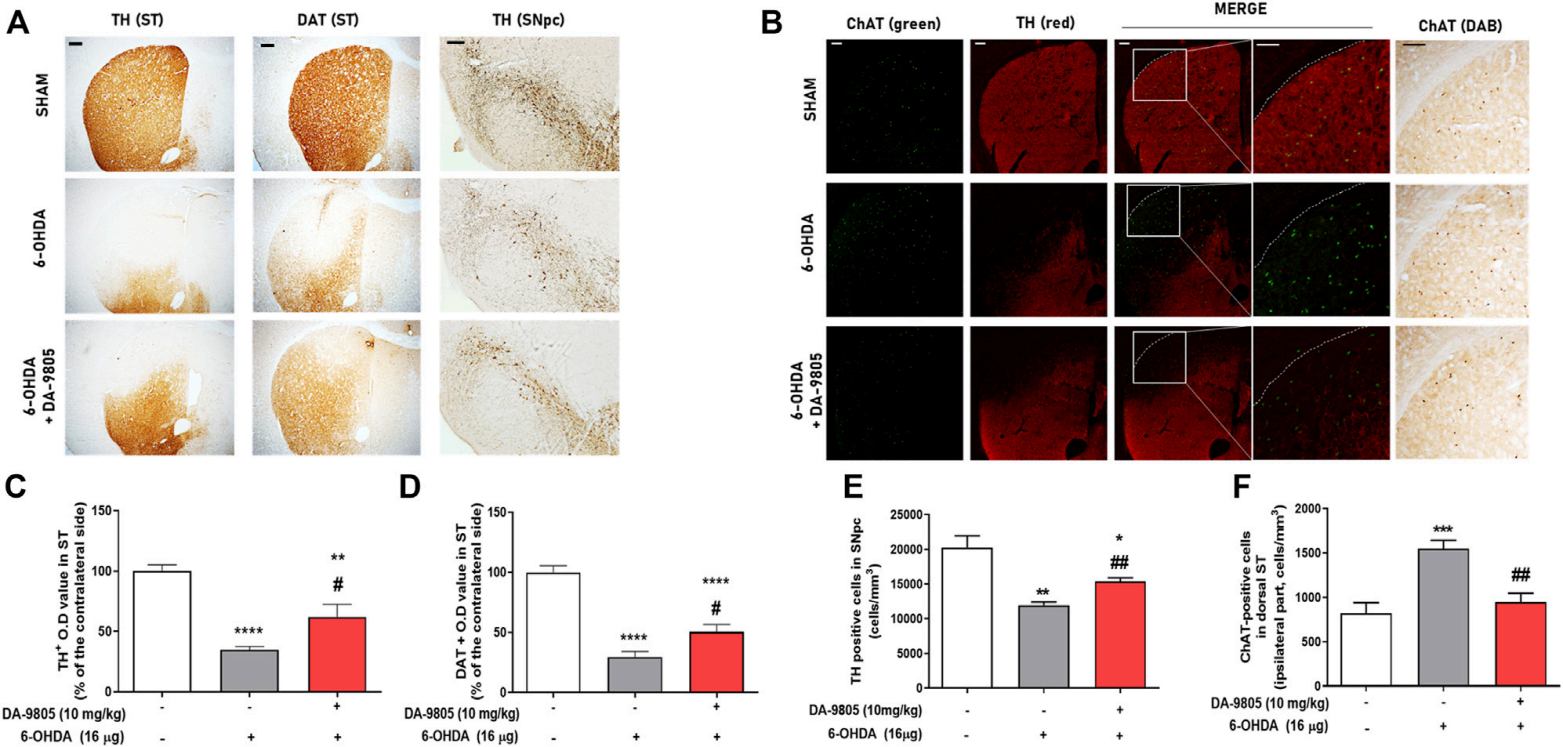
Effect of DA-9805 on enzymes for dopamine-acetylcholine homeostasis in 6-hydroxydopamine (6-OHDA)-lesioned mouse dorsal striatum (ST) and substantia nigra pars compacta (SNpc). Representative photographs of tyrosine hydroxylase (TH) and dopamine transporter (DAT) staining **(A)**, dual label fluorescence of Choline transferase (ChAT) and TH **(B)**. Graphs were represented as optical density value of TH or DAT-positive fibers **(C,D)** in the dorsal ST, the number of TH positive cells **(E)** in the SNpc and the number of ChAT positive cells **(F)** in the dorsal ST. Scale bar = 100 μm. Values are given as the mean ± S.E.M. **(C–F)** Data were analyzed by One-way ANOVA followed by post hoc Bonferroni’s multiple comparisons test. **p < 0.01 and ****p < 0.0001; compared to the sham group. ^#^ *p* < 0.05; compared to the 6-OHDA group. N = 6 per group.

We aimed to determine whether DA-9805 could affect neurotransmitter regulators of both DA and Ach in ST. First, we investigated the effects of DA-9805 on expression levels of TH and DAT in the dorsal ST of 6-OHDA-injected mouse brains. We observed that 6-OHDA significantly decreased the expression of TH in ST compared to the sham (by 34.85 ± 2.68%). However, DA-9805 increased the TH expression in ST (178.10 ± 29.83% compared to the 6-OHDA group) (Figures 3A,C). Furthermore, 6-OHDA significantly reduced DAT expression in the ST compared to the sham (by 29.61 ± 4.62%) while DA-9805 significantly up-regulated DAT expression in the ST against the 6-OHDA toxicity (170.97 ± 20.07%) (Figures 3A,D). We also measured the effects of DA-9805 on expression levels of TH in the SNpc of 6-OHDA-injected mouse brains. 6-OHDA induced dopaminergic neuronal cell death in the SNpc, which decreased by 58.93% compared to the sham (11953 ± 468.40 cells/mm^3^ in 6-OHDA group and 20282 ± 1660.00 cells/mm^3^ in the sham group). However, DA-9805 significantly inhibited the damage by 128.36% of the SNpc of 6-OHDA-injected mouse (15343 ± 561.50 cells/mm^3^ in DA-9805 group) (Figures 3A,E).

To elucidate whether DA-9805 regulated the expression of striatal ChAT, we used a transferase enzyme for the synthesis of Ach. Since the cholinergic interneurons were in the dorsal ST (Dorst et al., 2020), we analyzed the expression of ChAT in the dorsal part of the lesioned side of ST. Administration of 6-OHDA was associated with a significant increase in the number of ChAT in the dorsal part of the ST (1547.00 ± 95.53 cells/mm^3^), by 188.27% compared to Sham group (821.90 ± 118.50 cells/mm^3^). However, DA-9805 reverted the expression of ChAT in ST by 61.22% compared with that of the 6-OHDA group (947.50 ± 100.10 cells/mm^3^) (Figures 3B,F). These outcomes suggest that DA-9805 treatment affected to the striatal levels of neurotransmitters in 6-OHDA-lesioned ST by modulating TH, DAT and ChAT expression levels.

### 3.4 Ach has a Moderate Correlation With Effects of DA-9805 on Motor Functions and Striatal Neurotransmitters

**FIGURE 4:**
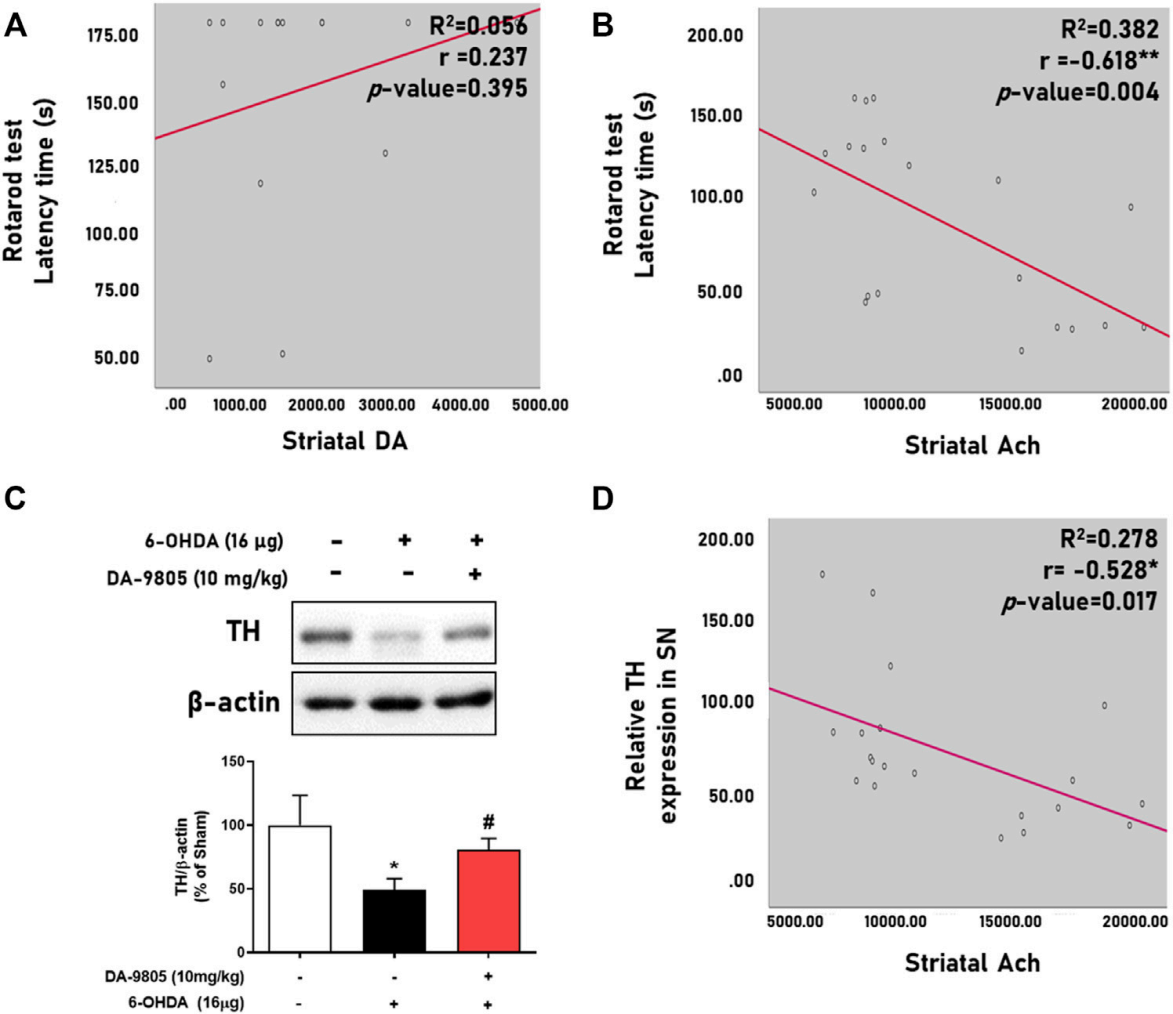
Correlation between levels of striatal neurotransmitters and phenotypes of Parkinson’s disease. Linear regression graph of latency time in rotarod test and striatal dopamine (DA) or acetylcholine (Ach) **(A,B)**, representative band image of tyrosine hydroxylase (TH) expression in the substantia nigra (SN) and their intensities were shown by the graph **(C)**. Linear regression of the relative TH expression in SN and striatal Ach **(D)**. N = 4 per group for Figure 4A and N = 4 in Sham group and N = 8 in 6-OHDA and DA-9805 groups for Figures 4B–D.

We evaluated the relationship between the neurotransmitters and motor functions by analyzing their correlation. We found that striatal Ach displayed a significantly moderate negative correlation with the latency time in the rotarod test (Figure 4B) rather than DA (Figure 4A) and other neurotransmitters (Table 1). Additionally, we tried to evaluate the relation between the striatal Ach level and dopaminergic neuronal death for highlighting the balance of DA-Ach systems. Since the levels of DA or Ach were investigated with different striatal tissues, we measured the TH expression in the symmetric region of the SN of mouse used for Ach measurement and analyzed correlation between them. Similar to the result in Figure 3E, Figure 4C showed the significant effect of DA-9805 on 6-OHDA-induced dopaminergic neuronal cell death (6-OHDA group, 49.60%; DA-9805 group, 80.83% compared to the sham group, respectively). Moreover, we found that only the levels of striatal Ach demonstrated a significant correlation with TH in the SN (Figure 4D). These findings demonstrated that the effects of DA-9805 demonstrated a significant degree of association with the suppressing striatal Ach levels, which showed a moderately negative correlation with TH expression in the SN.

**TABLE 1:** Pearson correlation (r) between striatal neurotransmitters and phenotypes of PD.

Measurements		Striatal neurotransmitter	
		GABA	Glu
Latency time (Rotarod test)	Pearson Correlation	0.169	−0.297
	*p*-value	0.476	0.204
TH expression in SN	Pearson Correlation	0.121	−0.346
	*p*-value	0.611	0.136

GABA, γ-aminobutyric acid; Glu, glutamate; TH, tyrosine hydroxylase; SN, substantia nigra.

**Correlation is significant at the 0.01 level (2-tailed).

## 4 Discussion

This study demonstrated that post-treatment with DA-9805 may restore 6-OHDA-induced motor deficits and imbalance of striatal neurotransmitters. Striatal neurotransmitters play a pivotal role as chemical messengers in regulating the motor functions. Disruption of the balance between the direct and indirect pathways associated with the striatal neurons results in the progression of movement disorder in PD (Dauer and Przedborski, 2003). Therefore, it is imperative to elucidate and control the alteration of striatal neurotransmitters to treat PD. Additionally, 6-OHDA has been associated with production of an extremely oxidizable toxicity, which facilitates the selective damage of catecholaminergic neurons (Su et al., 2018; Zeng et al., 2018). Administration of 6-OHDA into ST results in gradual and partial damage to the structure of nigrostriatal DA pathway for 4 weeks, following which it subsequently alters other striatal neurotransmitters (Alvarez-Fischer et al., 2008; Duty and Jenner, 2011). In the present study, we orally administered DA-9805 with a dose-dependent manner to mice daily for 10 days, 1 week after 6-OHDA injection. Post-treatment with DA-9805 recovered 6-OHDA-induced motor deficits in the pole, rotation or rotarod tests. These results indicated that DA-9805 effectively attenuated 6-OHDA-induced motor dysfunctions (Figure 1). In this study, DA-9805 was administered as the 6-OHDA-induced neurodegeneration progressed. Therefore, it can be interpreted that the efficacy of DA-9805 was viewed as a neuroprotection, but also acted on other mechanisms. As a result of confirming the expression of dopaminergic neurons projected in the nigrostriatal pathway through tissue analysis in the brain, it was increased by about 1.3 times compared to the 6-OHDA-treated mouse SNpc when DA-9805 was administered, whereas in the case of DAergic fibers decreased by 6-OHDA in the ST region, DA-9805 administration increased them about double. It shows a similar trend in the results of DAT, indicating the possibility of boosting fibers (Figure 3). According to a previous study, Bupleurum falcatum and its active compound, saikosaponin, were found to have neuroregenerative effects in spinal cord injury (Zhou et al., 2014; Huang et al., 2018; Ahmadimoghaddam et al., 2021). However, further study is needed to report whether it can act specifically on neuronal fibers.

Characteristically, neurons are activated and suppressed by Glu and GABA or Ach, respectively. Neurotransmitters have been pathologically altered in PD condition with a decrease of the striatal DA and GABA levels and an increase of the striatal Glu and Ach levels (Barone, 2010; Jamwal and Kumar, 2019). We investigated what neurotransmitters were influenced by the effects of DA-9805 in PD state. Decrease in the levels of striatal DA and GABA and increase in the levels of Glu and Ach was noted in the 6-OHDA group (Figure 2). This result demonstrates a similar tendency to that noted in PD patients. However, DA-9805-induced recovery of the motor dysfunctions was only associated with a tendency to increase DA levels, which was particularly similar to the results of a previous study with MPTP-induced PD mice (Jeong et al., 2018). However, striatal Ach showed the strongest correlation with rotarod latency times (Table 1 and Figure 2B). This outcome suggested that DA-9805 could attenuate motor dysfunctions by inhibiting increased Ach levels instead of supplementing DA in PD mice. In this study, it was hard to analyse the correlation between DA and Ach directly and we analysed their correlation by measuring striatal Ach level and TH expression in SN region. Although results show that Ach and expression of TH in SN were negatively correlated, we should careful to interpret it since TH expression of SN might not reflect the that of SNpc. Thus, it is needed to be additional analysed striatal DA and Ach simultaneously for future study.

Studies have reported that the levels of DA are induced by activation of nicotinic Ach receptors (nAchRs) and muscarinic Ach receptors (mAChR). M2-like receptors (M2 and M4) are involved in inhibiting the excitatory pathway, which is similar to D2 dopamine-receptor like receptors (D2 and D4) (Wess et al., 2007; Ztaou et al., 2016). Automatic inhibition of Ach release activity by the M4 receptor was ameliorated by an increase in regulator of G protein signalling 4 protein in cases of PD (Moehle et al., 2017). Cholinergic neurons continue to release ACh in an uncontrolled state, without the (negative) feedback by the M4 receptor; furthermore, increased striatal Ach influence the indirect pathways and results in motor or non-motor symptoms associated with PD (Sun et al., 2012). In addition, dopaminergic neurons appear to postpone the cholinergic interneurons firing, suggesting that dopaminergic signals, especially of D2 receptor, could directly inhibits cholinergic transmission (Schulz and Reynolds, 2013). In this study, we found the alterations in the levels of striatal neurotransmitters, especially of Ach, by DA9805 and it could be interpreted as being controlled by the dopaminergic neuroprotective effect of DA-9805.

There are several regulatory factors in ST that are directly associated with the DA or Ach release. TH, a rate-limiting enzyme of catecholamine biosynthesis, and DAT, a transmembrane transporter, are responsible for the regulating DA release and transmission (Rizzi and Tan, 2017). ChAT is involved in Ach synthesis, and is responsible for the cholinergic systems. According to outcomes presented in Figure 3, the expression of TH and DAT in the 6-OHDA group were lower than those of the sham group, contrary to the increased ChAT expression in ST. Furthermore, we presented that the effects of DA-9805 on the levels of striatal Ach were significantly moderate in correlation with TH in SN (Figure 3D). Therefore, DA-9805 could be used to reduce the damage of the factors associated with DA transmission, while suppressing the amount of Ach by inhibiting the enzymes involved in Ach synthesis. However, this study has some limitations to declare the regulatory effects of DA-9805 on striatal neurotransmitter metabolic pathways since we did not evaluate the upstream factors of pathway such as tyrosine, phenylalanine or choline. Thus, further study to elucidate striatal DA and Ach metabolic pathway regulated by DA-9805 should be needed.

In summary, the current study demonstrated that DA-9805 may have therapeutic effects on the motor deficits of PD during the progression of 6-OHDA-induced loss of DA. Effects of DA-9805 administration were closely related to the reduction in the amount of striatal Ach levels by ChAT suppression. Additionally, it was also associated with DA transmission in striatal DA pathway by maintaining the balance of DA and Ach levels in PD state as well as the neuroprotection. Therefore, this study supports the fact that DA-9805 can be a potential therapeutic agent for PD.

## Data Availability

The original contributions presented in the study are included in the article/Supplementary Materials, further inquiries can be directed to the corresponding author.
