# Supplementary material for: DA-9805, a Herbal Mixture, Restores Motor Manifestations in 6-Hydroxydopamine-induced Parkinson’s Disease Mouse Model by Regulating Striatal Dopamine and Acetylcholine Levels
Source: Front Pharmacol. 2022 Jun 15;13:903664. doi: 10.3389/fphar.2022.903664 (PMC9240257; doi:10.3389/fphar.2022.903664)
Supplement: Supplementary file 1 [file Presentation1.PPTX]

## Slide 1
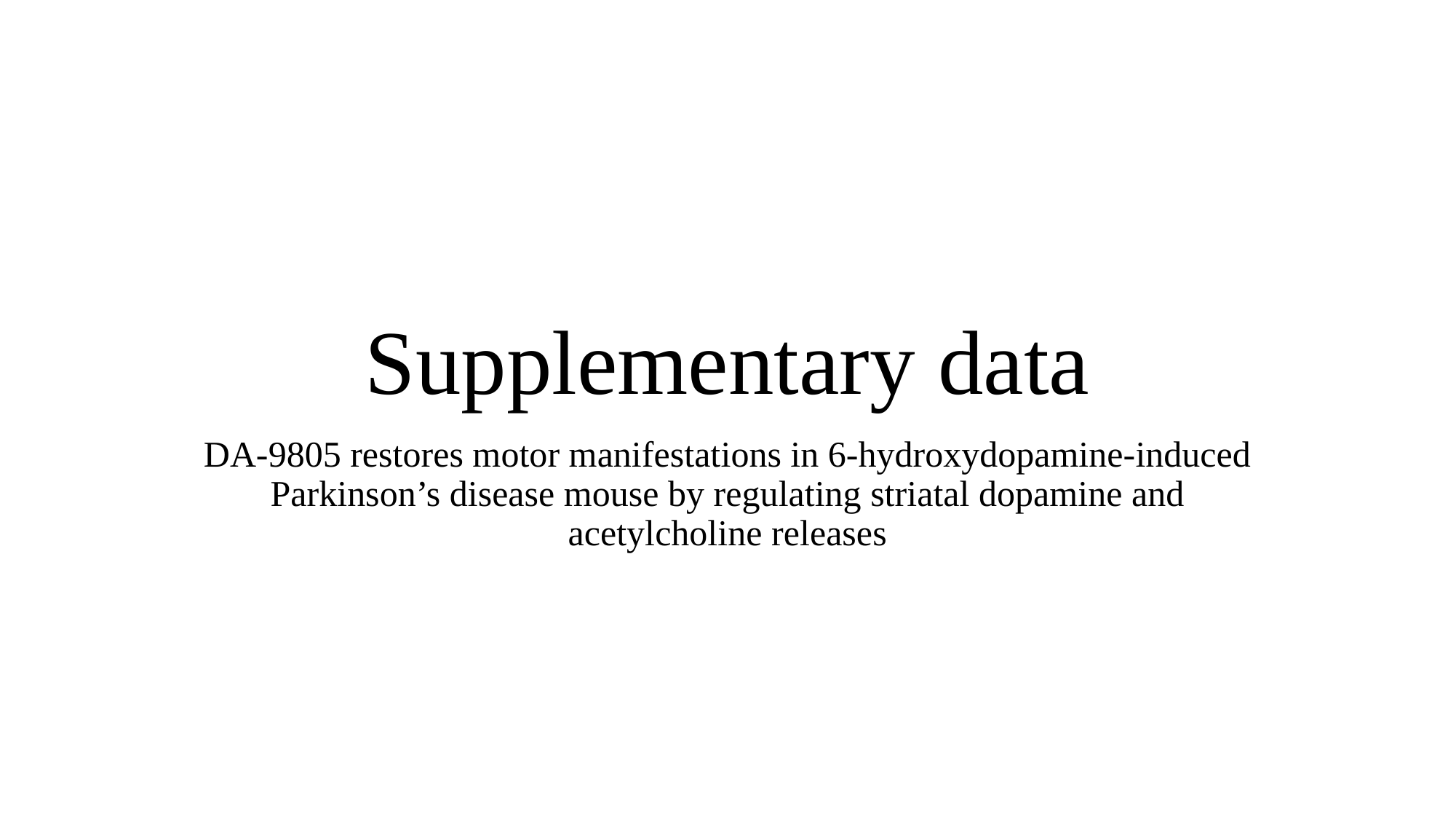

# Supplementary data
DA-9805 restores motor manifestations in 6-hydroxydopamine-induced Parkinson’s disease mouse by regulating striatal dopamine and acetylcholine releases

## Slide 2
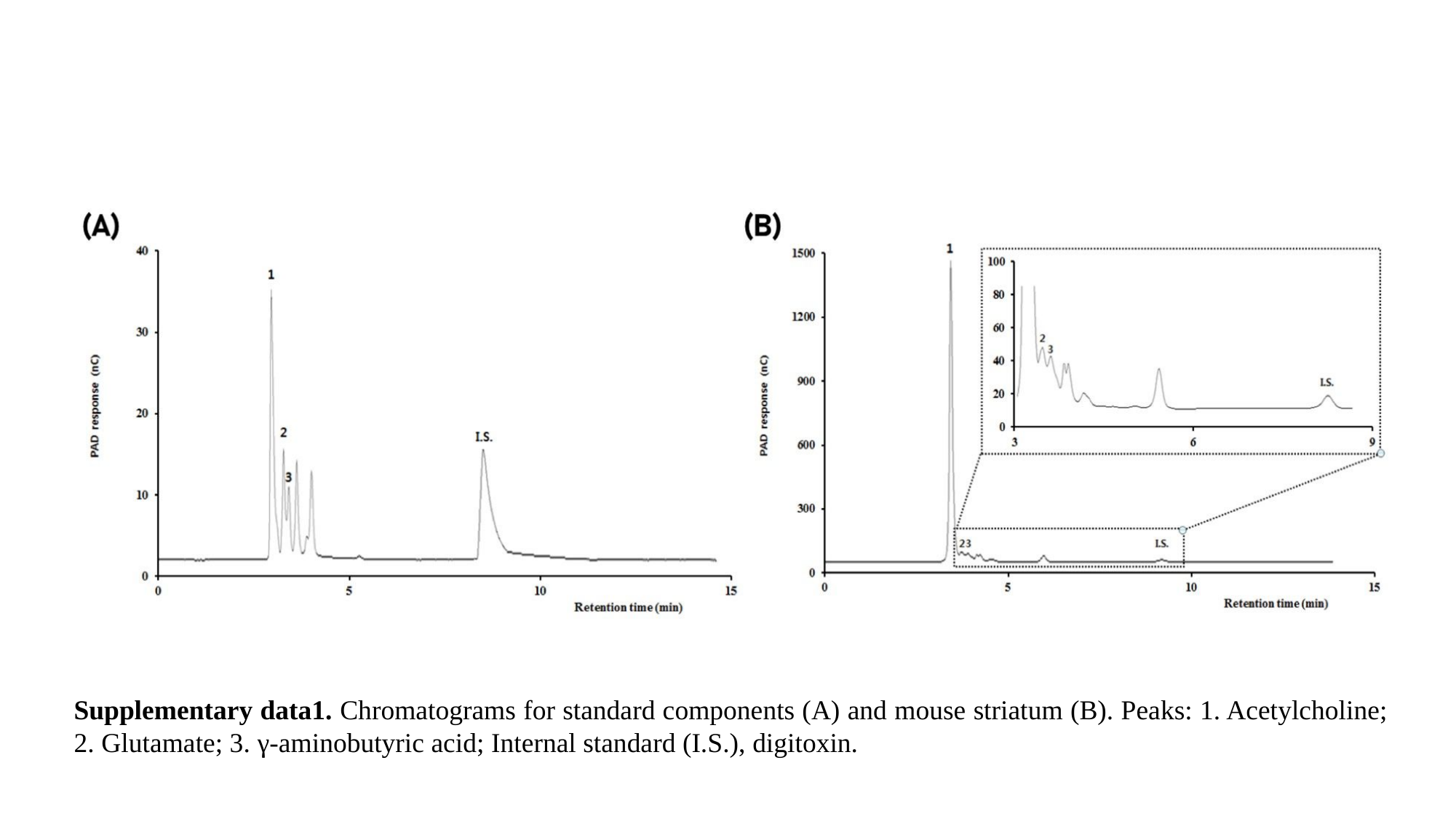

Supplementary data1. Chromatograms for standard components (A) and mouse striatum (B). Peaks: 1. Acetylcholine; 2. Glutamate; 3. γ-aminobutyric acid; Internal standard (I.S.), digitoxin.

## Slide 3
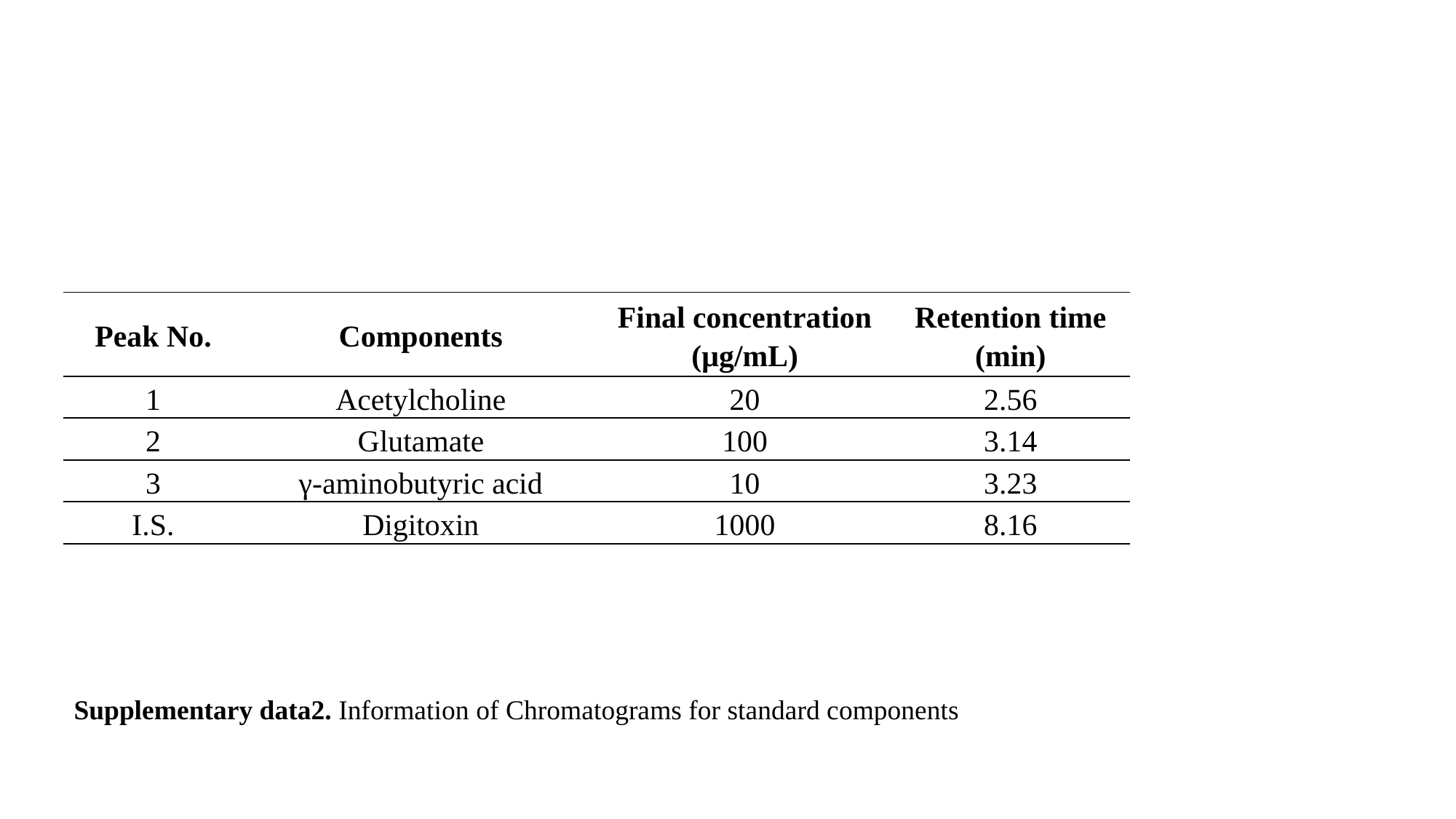

| Peak No. | Components | Final concentration (μg/mL) | Retention time (min) |
| --- | --- | --- | --- |
| 1 | Acetylcholine | 20 | 2.56 |
| 2 | Glutamate | 100 | 3.14 |
| 3 | γ-aminobutyric acid | 10 | 3.23 |
| I.S. | Digitoxin | 1000 | 8.16 |
Supplementary data2. Information of Chromatograms for standard components
